# Associations of the triglyceride-glucose index and remnant cholesterol with coronary artery disease: a retrospective study

**DOI:** 10.1186/s12944-024-02036-w

**Published:** 2024-02-10

**Authors:** Xiaosheng Wu, Weiping Qiu, Houlin He, Guojun Zhao, Jianling Liu

**Affiliations:** 1 Affiliated Qingyuan Hospital, Guangzhou Medical University (Qingyuan People’s Hospital), Qingyuan Guangdong, China; 2 Guangzhou Medical University, Guangzhou Guangdong, China

**Keywords:** Triglyceride-glucose index, Remnant cholesterol, Coronary artery disease, Insulin resistance

## Abstract

**Background:**

Remnant cholesterol (RC) represents a low-cost and readily measured lipid index that contributes significantly to residual cardiovascular disease risk. The triglyceride-glucose (TyG) index exhibits a significant correlation with cardiovascular disease occurrence. However, RC and the TyG index have rarely been examined for their potentials in predicting coronary artery disease (CAD). Accordingly, the study was designed to validate the correlations of these two biomarkers with CAD and to compare the forecasted values of these two biomarkers for newly diagnosed CAD.

**Methods:**

Totally 570 subjects firstly administered coronary angiography were enrolled, including 431 newly diagnosed CAD cases and 139 individuals without CAD. The individuals were classified into two groups according to CAD diagnosis. RC was derived as total cholesterol content (mmol/L) – (high density lipoprotein cholesterol content + low density lipoprotein cholesterol content; both in mmol/L). The TyG index was determined as ln (fasting triglyceride level [mg/dL] × fasting plasma glucose level [mg/dL])/2.

**Results:**

Baseline feature analysis revealed significant differences in RC and the TyG index between the CAD and non-CAD groups (both *P* < 0.001). RC and the TyG index were independent risk factors for CAD in accordance with logistic regression analysis (both *P* < 0.05). Moreover, spearman correlation analysis elucidated CAD had a more remarkable correlation with the TyG index compared with RC (both *P* < 0.001). Furthermore, according to receiver operating characteristic curve analysis, the TyG index was better than RC in predicting CAD.

**Conclusions:**

The TyG index and RC have significant associations with CAD. Compared with RC, the TyG index possesses a closer correlation with CAD and a higher predictive value for CAD.

## Introduction

Coronary artery disease (CAD), a chronic cardiac disorder triggered by the narrowing of coronary arteries, represents the top global contributor to mortality, with nearly half of all fatalities attributed to CAD [1, 2]. CAD is also correlated with elevated burden of inflammation [3]. Similarly, inflammation is a characteristic feature in conditions highly promoting CAD such as metabolic syndrome [4], hypertension [5], type 2 diabetes mellitus (T2DM) [6], non-alcoholic fatty liver disease [7], obesity [8], and diabetic nephropathy [9]. Some individuals with sudden cardiac death resulting from CAD have no clinical symptoms; however, it was only discovered by autopsy that such individuals previously had severe CAD [10]. Catheter-based invasive coronary angiography (CAG), considered the ultimate tool for CAD diagnosis, effectively ascertains both the degree and number of coronary artery stenoses. Guided by CAG findings, patients with ≥ 50% lumen constriction in a major coronary artery are diagnosed with CAD [11]. Yet, in the early stages of the disease, numerous patients refuse CAG due to its costly and invasive nature, as well as its potential for serious complications. Patients’ refusal results in missed accurate disease evaluation and timely coronary revascularization. In addition, CAD cases are at high risk of recurrence. Thus, the development of a straightforward and practical biomarker to predict CAD is urgent.

Remnant cholesterol (RC), the cholesterol level of triglyceride-rich lipoproteins, significantly contributes to cardiovascular disease (CVD) development [12–14]. RC can permeate arterial walls, accumulate within the intima and promote the generation of foam cells and atherosclerotic plaques [15–17]. Moreover, RC can induce arterial inflammation alongside cellular immune reactions [18]. Consequently, RC can be readily computed from the lipid profile employing a formula, providing invaluable clinical insights without additional expenditure.

The triglyceride-glucose (TyG) index contributes significantly to CVD progression and prognosis [19–22]. The TyG index is considered a dependable and newfound biomarker for gauging insulin resistance (IR) [22, 23]. Pathological and physiological studies have shown IR induces inflammatory reactions, dyslipidemia and vascular endothelial dysfunction, which might constitute the main mechanism of CVD progression [24]. Mounting evidence suggests that triglyceride-based indexes are also associated with diseases related to chronic inflammation, including T2DM [25], hepatosteatosis [26], hypertension [27], and cardiac conditions [28].

RC and the TyG index are risk elements for CVD [12–14, 20, 21], but few reports have specifically explored the associations of these elements with CAD at first diagnosis, and no studies have compared their predictive powers in this patient population. Understanding the associations of RC and the TyG index with CAD as well as their predictive values in CAD could help identify individuals at high risk of CAD early, serving as auxiliary screening indicators for invasive CAG, and enhancing the understanding of pathophysiological mechanisms. Accordingly, the investigation aimed to validate the correlations of the TyG index and RC with CAD at first diagnosis, and to compare their predictive powers in CAD.

## Method

### Ethical statements

This retrospective study had approval from the Ethics Committee of the Affiliated Qingyuan Hospital, Guangzhou Medical University (Qingyuan People's Hospital) (IRB-2023–003). The requirement for informed consent was waived owing to the retrospective nature of the study.

### Study design

**Fig. 1:**
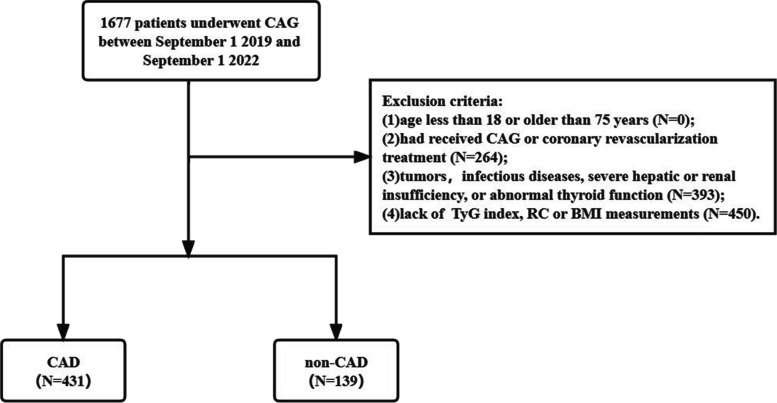
Flow chart of subject recruitment. CAG, coronary angiography; CAD, coronary artery disease; TyG, triglyceride-glucose; RC, remnant cholesterol; BMI, body mass index

The current study reviewed 1677 patients who underwent first CAG in the Affiliated Qingyuan Hospital, Guangzhou Medical University (Qingyuan People's Hospital) between September 1, 2019, and September 1, 2022. Exclusion conditions were: (1) age below 18 years or above 75 years; (2) previous CAG or coronary revascularization therapy; (3) malignancies, infectious diseases, severe hepatic or renal insufficiency, or impaired thyroid function; and (4) incomplete TyG index, RC or body mass index (BMI) measurements. Ultimately, 570 subjects were recruited, comprising 431 newly diagnosed CAD cases and 139 patients without CAD (Fig. 1). In accordance with the established diagnostic criteria for CAD, the individuals were classified into the CAD (*n* = 431) as well as non-CAD (*n* = 139) groups.

### Data source and collection

Patient data were compiled from the autonomous digital medical record system, including key demographic features, clinical background, outcomes of blood analysis, and pertinent medical imaging findings. Demographic characteristics encompassed age, gender, weight, height, blood pressure, and smoking and alcohol consumption habits. Clinical history embraced hypertensive and diabetic medical history. The use of antihypertensive, antidiabetic and antilipidemic drugs were also analyzed.

The blood specimens subjected to examination were obtained in the morning through routine collection of fasting venous blood by skilled medical professionals. Total cholesterol (TC), triglyceride (TG), high-density lipoprotein cholesterol (HDL-C), low-density lipoprotein cholesterol (LDL-C), fasting plasma glucose (FPG) and glycosylated hemoglobin (HbA1c) were assessed on an automated hematology analyzer. Catheter-based invasive CAG was performed by percutaneous radial or femoral arteriography. The employed angiography apparatus accurately diagnosed all manifestations of coronary arteries.

### Definitions

CAD is characterized by narrowing of ≥ 50% in one principal coronary artery [11]. CAD severity is gauged by the count of vessels displaying narrowing of ≥ 50%. Per the guideline of the World Health Organization on diabetes, the criteria for diagnosing T2DM are outlined as follows: FPG ≥ 7.0 mmol/L; 2-h plasma glucose content ≥ 11.1 mmol/L based on the oral glucose tolerance test; HbA1c ≥ 6.5%; or a documented history of T2DM [29]. The TyG index was computed as ln (TG [mg/dL] × FPG [mg/dL])/2 [30]. RC was derived as follows: RC = TC (mmol/L) – HDL-C (mmol/L) – LDL-C (mmol/L) [31].

### Statistical analysis

Continuous variates were shown as median and interquartile range (IQR) encompassing the 25th and 75th percentiles, which were compared among various groups using the Kruskal–Wallis test. Categorical variates were presented in the form of count or percentage, which were compared across the groups using the chi-square test.

Logistic regression models, determining the odds ratio (OR) and the corresponding 95% confidence interval (CI) for each parameter, were established to validate the links of RC and the TyG index (independent variables) with CAD (dependent variable). Starting with RC quartiles, three distinct models were generated to validate the correlation between RC and CAD, accounting for significant covariates: Model 1, an unaltered state; Model 2, refinement by the inclusion of age and sex; Model 3, fine-tuning by adjustment of variables in Model 2, as well as systolic blood pressure (SBP), BMI, current smoking and drinking habits, HbA1c, T2DM, hypertension, antihypertensive drugs, antidiabetic drugs and antilipidemic drugs. Similarly, within TyG index quartiles, three different models were established: Model 1, an unaltered state; Model 2, refinement by the inclusion of age and sex as covariates; Model 3, refinement by adjustment of variables in Model 2, along with SBP, BMI, current smoking and drinking, hypertension, antihypertensive drugs, antidiabetic drugs and antilipidemic drugs.

The connections of the TyG index and RC with CAD were elucidated through the use of spearman correlation analysis. Furthermore, the predictive efficacy for CAD was evaluated through the use of receiver operating characteristic (ROC) curve as well as the determination of the area under the curve (AUC) and the corresponding 95% CIs. *P* < 0.05 was deemed statistically significant. SPSS 26.0 (IBM, USA) and GraphPad Prism 8.0 (GraphPad Software, USA; www.graphpad.com) were utilized for statistical analysis.

## Results

### Clinicodemographic features of the non-CAD and CAD groups

Clinicodemographic analysis involved 570 subjects, with 431 patients newly diagnosed with CAD and 139 individuals without CAD (Table 1). Mean patient age was 51 years (IQR, 43–54). There were 484 men (84.9%). Within these two groups, variables such as age, sex, smoking history, HbA1c, RC, TyG index, T2DM history, and use of antidiabetic drugs exhibited marked differences (all *P* < 0.05). Meanwhile, within these two groups, variables encompassing SBP, diastolic blood pressure (DBP), BMI, history of alcohol consumption, and use of antihypertensive drugs and antilipidemic drugs had no discernible differences (all *P* > 0.05).

**Table 1:** Clinicodemographic features of the non-CAD and CAD groups

	**non-CAD**	**CAD**	***P value***
N	139	431	
Age (year)	51 (43, 54)	51 (46, 55)	0.041
Male, n (%)	98 (70.5)	386 (89.6)	< 0.001
SBP (mmHg)	130 (122, 148)	133 (119, 148)	0.662
DBP (mmHg)	85 (73, 95)	86 (76, 96)	0.564
BMI (kg/m2)	24.91 (22.86, 27.01)	24.91 (23.03, 27.34)	0.749
Smoking	41 (29.5%)	275 (63.8%)	< 0.001
Drinking	25 (18.0%)	92 (21.3%)	0.394
HbA1c (%)	5.8 (5.6, 6.0)	6.0 (5.7, 6.7)	< 0.001
RC (mmol/L)	0.30 (0.11, 0.55)	0.45 (0.24, 0.74)	< 0.001
TyG index	1.81 (1.66, 2.05)	2.03 (1.84,2.25)	< 0.001
Hypertension, n (%)	53 (38.1)	191 (44.3)	0.200
T2DM, n (%)	15 (10.8)	113 (26.3)	< 0.001
Antihypertensive drugs (n, %)	33 (23.7)	111 (25.8)	0.635
Antidiabetic drugs (n, %)	7 (5)	48 (11.1)	0.034
Antilipidemic drugs (n, %)	33 (23.7)	80 (18.6)	0.183

*CAD* Coronary artery disease, *SBP* Systolic blood pressure, *DBP* Diastolic blood pressure, *BMI* Body mass index, HbA1c, glycosylated hemoglobin, *RC* remnant cholesterol, *TyG* Triglyceride-glucose, *T2DM* Type 2 diabetes mellitus

### Associations of RC and the TyG index with CAD

RC was stratified into four tiers based on quartile: I (0 ≤ RC < 0.1976), II (0.1976 ≤ RC < 0.42), III (0.42 ≤ RC < 0.70), and IV (0.7 ≤ RC ≤ 8.38). As shown in Table 2, the results demonstrated a notable correlation between RC and CAD after multivariate adjustment (*P* < 0.05). With RC as a continuous variable, a significant correlation with CAD was firmly established (OR = 1.790, 95%CI 1.108–2.894, *P* < 0.05). With RC as a categorical variate, CAD risk was 2.178 fold higher in cases categorized as IV compared with category I cases (95%CI 1.117–4.246, *P* < 0.05).

**Table 2:** Association between RC and CAD

Variables	Coronary artery disease					
	**Model 1**		**Model 2**		**Model 3**	
	**OR (95% CI)**	***P value***	**OR (95% CI)**	***P value***	**OR (95% CI)**	***P value***
RC	1.947(1.218–3.113)	0.005	1.833(1.135–2.960)	0.013	1.790(1.108–2.894)	0.017
I	Reference		Reference		Reference	
II	1.440(0.873–2.377)	0.153	1.352(0.798–2.290)	0.263	0.977(0.543–1.759)	0.939
III	2.011(1.183–3.417)	0.010	1.785(1.024–3.112)	0.041	1.467(0.802–2.684)	0.213
IV	3.490(1.928–6.318)	< 0.001	3.257(1.760–6.026)	< 0.001	2.178(1.117–4.246)	0.022
*P-trend*	< 0.001		< 0.001		0.011	

Model 1: unadjusted

Model 2: adjusted for age and sex

Model 3: adjusted for age, sex, SBP, BMI, smoking, drinking, HbA1c, T2DM, hypertension, antihypertensive drugs, antidiabetic drugs and antilipidemic drugs

*RC* Remnant cholesterol, *CAD* coronary artery disease, *SBP* systolic blood pressure, *BMI* body mass index, *HbA1c* glycosylated hemoglobin, *T2DM* Type 2 diabetes mellitus

Likewise, the TyG index was distributed across four groups based on quartile: I (1.17 ≤ TyG ≤ 1.77), II (1.77 < TyG ≤ 1.99), III (1.99 < TyG ≤ 2.22), and IV (2.22 < TyG ≤ 3.89). Logistic regression models unveiled a prominent linkage of the TyG index with CAD (*P* < 0.001, Table 3). With the TyG index as a continuous variate, a notable connection with CAD was demonstrated (OR = 1.056, 95%CI 1.027–1.086, *P* < 0.001). In addition, with the TyG index as a categorical variate, CAD risk levels were 2.594 (95%CI 1.472–4.570, *P* = 0.001), 3.474 (95%CI 1.889–6.389, *P* < 0.001), and 6.419 (95%CI 3.203–12.865, *P* < 0.001) fold higher in patients categorized as II, III, and IV cases, respectively, versus category I cases after adjustment for confounders.

**Table 3:** Association between the TyG index and CAD

Variables	Coronary artery disease					
	**Model 1**		**Model 2**		**Model 3**	
	**OR (95% CI)**	***P value***	**OR (95% CI)**	***P value***	**OR (95% CI)**	***P value***
TyG index	7.641(3.919–14.899)	< 0.001	7.699(3.877–15.288)	< 0.001	1.056(1.027–1.086)	< 0.001
I	Reference		Reference		Reference	
II	2.995(1.795–4.995)	< 0.001	2.839(1.659–4.861)	< 0.001	2.594(1.472–4.570)	0.001
III	3.622(2.114–6.206)	< 0.001	3.420(1.946–6.013)	< 0.001	3.474(1.889–6.389)	< 0.001
IV	6.510(3.517–12.051)	< 0.001	6.740(3.554–12.782)	< 0.001	6.419(3.203–12.865)	< 0.001
*P-trend*	< 0.001		< 0.001		< 0.001	

Model 1: unadjusted

Model 2: adjusted for age and sex

Model 3: adjusted for age, sex, SBP, BMI, smoking, drinking, hypertension, antihypertensive drugs, antidiabetic drugs and antilipidemic drugs

*TyG* triglyceride-glucose, *CAD* Coronary artery disease, *SBP* Systolic blood pressure, *BMI* Body mass index

Spearman correlation analysis indicated significant associations of CAD with RC (*r* = 0.182, *P* < 0.001) and the TyG index (*r* = 0.271, *P* < 0.001).

### Predictive values of the RC and the TyG index in CAD

**Fig. 2:**
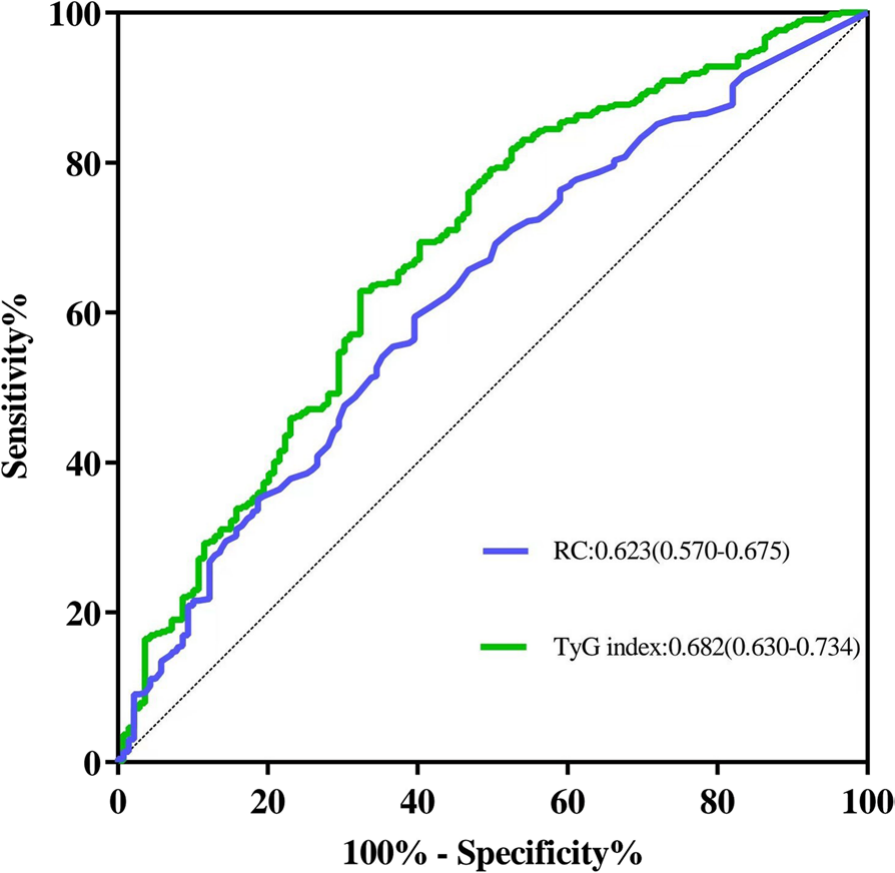
ROC curve analysis of RC and the TyG index for CAD prediction. ROC, receiver operating characteristic; RC, remnant cholesterol; TyG, triglyceride-glucose; CAD, coronary artery disease

ROC curve analysis of RC and the TyG index for CAD prediction is shown in Fig. 2. The AUC of the TyG index for CAD prediction was 0.682 (95%CI 0.630–0.734, *P* < 0.001), which significantly surpassed that of RC at 0.623 (95%CI 0.570–0.675, *P* < 0.001). The data document that both RC and the TyG index have promising predictive performances for CAD, with the TyG index showing superiority over RC.

## Discussion

This retrospective study demonstrated remarkable associations of the TyG index and RC with CAD, which were independent of traditional cardiovascular risk factors. Moreover, this study firstly revealed the TyG index as a parameter more tightly related to CAD, with a heightened predictive capability for CAD, compared with RC.

This investigation unveiled a substantial and meaningful correlation between RC and CAD occurrence. A previous study assessing 1716 individuals with acute coronary syndrome administered percutaneous coronary intervention revealed that heightened RC (> 0.79 mmol/L) was associated with CVD [32]. This finding corroborated the present study, suggesting that RC contributed to the residual cardiovascular risk in CAD cases. A cohort study including 4331 CAD patients revealed that high RC was closely associated with unfavorable outcomes in both diabetic and prediabetic CAD patients [33]. The propensity of RC to infiltrate the arterial walls and accumulate within the intima, promoting the generation of foam cells, induces a cascade involving low-grade inflammation and vascular endothelial dysfunction, culminating in atherosclerosis ultimately [34]. Hence, the integration of RC assessment into routine clinical practice has paramount importance in mitigating residual cardiovascular risk.

Previous evidence indicated the TyG index possessed a robust impact on CVD risk among both diabetic and non-diabetic patients [35–37]. Corroborating previous findings, this study demonstrated the TyG index was a risk element for CAD. CAD severity is intrinsically related to the count of narrowed vessels. Recent studies reported that the TyG index had a linkage with CAD severity in T2DM cases, while such an association was not detected in individuals with normal glycometabolism [11, 38]. Since TyG index assessment considers TG and FPG, coupled with the outcomes emerging from the present study, it becomes evident that the TyG index contributes to CAD. Furthermore, the impact of the TyG index in relation to the count of stenosed vessels is possibly modulated by the effect of blood glucose.

Most significantly, this observation supported that the TyG index surpassed RC in terms of predictive power in CAD. The rationale can be attributed to IR. IR promotes endothelial impairment as well as atherogenesis initiation and progression. Furthermore, it is associated with the perturbation of the inflammatory milieu, thrombotic equilibrium, dyslipidemia, and hypertension, culminating in the stenosis of the coronary artery [36, 39, 40]. In agreement, IR increases very low-density lipoprotein production, resulting in escalated RC synthesis [34]. In clinical practice, alongside mitigating lipid residual risk, early detection and appropriate management of dysglycemia are expected to substantially affect the prognosis of patients with CAD concurrently undergoing active therapeutic interventions aimed at decreasing LDL-C. Moreover, the TyG index may be broadly applied in clinic to recognize individuals at high risk of CAD early.

### Strengths and limitations

The present study features a pioneering endeavor to compare the TyG index and RC for predictive efficacy in CAD. However, it is imperative to acknowledge that the exploration had limitations. First, causality could not be clearly established due to its retrospective design. Subsequent longitudinal studies may provide a more refined understanding in the future. Secondly, the relatively small size of the patient cohort might reduce the statistical robustness of the findings. Thirdly, the derivation of RC through calculation, while convenient, might not carry precision akin to direct measurement. Nevertheless, extensive findings underscored the parity between calculated and measured RC in predicting adverse cardiovascular events [32]. Fourthly, the potential effects of antilipidemic drugs on measurements and CAD occurrence could not be excluded [22]. Fifthly, this single-center study mainly included Chinese individuals, and the findings may not apply to broader populations. The generalizability of the outcomes require further exploration through large multicenter studies.

## Conclusion

The TyG index and RC had independent associations with newly diagnosed CAD. Furthermore, the TyG index exhibited a more substantial link with CAD and a higher predictive value for CAD than RC. These findings provide population-based evidence that assessing CAD risk can be achieved through TyG index monitoring, with significant implications for better understanding of the underpinning pathophysiological mechanisms. Most importantly, the TyG index, a low-cost, convenient and widely applicable biomarker, may be broadly employed in clinic to recognize individuals at high risk of CAD early, serving as an auxiliary screening indicator for invasive CAG and providing more accurate and effective monitoring as well as novel prevention strategies for the clinical management of CAD.

## Data Availability

Due to privacy and ethical limitations, the data generated and analyzed in the current study are not publicly available but can be obtained from corresponding authors upon reasonable request.

## Abbreviations

RC: Remnant cholesterol
TyG: Triglyceride-glucose
CAD: Coronary artery disease
T2DM: Type 2 diabetes mellitus
CVD: Cardiovascular disease
IR: Insulin resistance
CAG: Coronary angiography
BMI: Body mass index
TC: Total cholesterol
TG: Triglyceride
HDL-C: High-density lipoprotein cholesterol
LDL-C: Low-density lipoprotein cholesterol
FPG: Fasting plasma glucose
HbA1c: Glycosylated hemoglobin
IQR: Interquartile range
OR: Odds ratio
CI: Confidence interval
ROC: Receiver operating characteristic
AUC: Area under the curve
SBP: Systolic blood pressure
DBP: Diastolic blood pressure
